# Cardiac Magnetic Resonance as Risk Stratification Tool in Non-Ischemic Dilated Cardiomyopathy Referred for Implantable Cardioverter Defibrillator Therapy—State of Art and Perspectives

**DOI:** 10.3390/jcm12247752

**Published:** 2023-12-18

**Authors:** Adriana Argentiero, Maria Cristina Carella, Donato Mandunzio, Giulia Greco, Saima Mushtaq, Andrea Baggiano, Fabio Fazzari, Laura Fusini, Giuseppe Muscogiuri, Paolo Basile, Paola Siena, Nicolò Soldato, Gianluigi Napoli, Vincenzo Ezio Santobuono, Cinzia Forleo, Eduard Claver Garrido, Andrea Di Marco, Gianluca Pontone, Andrea Igoren Guaricci

**Affiliations:** 1 University Cardiology Unit, Interdisciplinary Department of Medicine, University of Bari Aldo Moro, 70121 Bari, Italy; adrianaargentiero92@gmail.com (A.A.); m.c.carella92@gmail.com (M.C.C.); d.mandunzio@studenti.uniba.it (D.M.); giulia-greco@libero.it (G.G.); pabas2304@gmail.com (P.B.); p.siena1@studenti.uniba.it (P.S.); nicolo.soldato@gmail.com (N.S.); gianluiginapoli@gmail.com (G.N.); eziosantobuono@gmail.com (V.E.S.); cinzia.forleo@uniba.it (C.F.); 2 Perioperative and Cardiovascular Imaging Department, Centro Cardiologico Monzino IRCCS, 20138 Milan, Italy; saima.mushtaq@cardiologicomonzino.it (S.M.); andrea.baggiano@cardiologicomonzino.it (A.B.); fabio.fazzari@cardiologicomonzino.it (F.F.); laura.fusini@cardiologicomonzino.it (L.F.); gianluca.pontone@cardiologicomonzino.it (G.P.); 3 Department of Radiology, ASST Papa Giovanni XIII, 24127 Bergamo, Italy; g.muscogiuri@gmail.com; 4 Bio-Heart Cardiovascular Diseases Research Group, Bellvitge Biomedical Research Institute (IDIBELL), L’Hospitalet de Llobregat, 08907 Barcelona, Spain; ecgarrido@bellvitgehospital.cat (E.C.G.); adimarco@bellvitgehospital.cat (A.D.M.); 5 Department of Cardiology, Hospital Universitari de Bellvitge, L’Hospitalet de Llobregat, 08907 Barcelona, Spain; 6 Department of Biomedical, Surgical and Dental Sciences, University of Milan, 20122 Milan, Italy

**Keywords:** dilated cardiomyopathy, sudden cardiac death, primary prevention, implantable cardioverter defibrillator, cardiac magnetic resonance

## Abstract

Non-ischemic dilated cardiomyopathy (DCM) is a disease characterized by left ventricular dilation and systolic dysfunction. Patients with DCM are at higher risk for ventricular arrhythmias and sudden cardiac death (SCD). According to current international guidelines, left ventricular ejection fraction (LVEF) ≤ 35% represents the main indication for prophylactic implantable cardioverter defibrillator (ICD) implantation in patients with DCM. However, LVEF lacks sensitivity and specificity as a risk marker for SCD. It has been seen that the majority of patients with DCM do not actually benefit from the ICD implantation and, on the contrary, that many patients at risk of SCD are not identified as they have preserved or mildly depressed LVEF. Therefore, the use of LVEF as unique decision parameter does not maximize the benefit of ICD therapy. Multiple risk factors used in combination could likely predict SCD risk better than any single risk parameter. Several predictors have been proposed including genetic variants, electric indexes, and volumetric parameters of LV. Cardiac magnetic resonance (CMR) can improve risk stratification thanks to tissue characterization sequences such as LGE sequence, parametric mapping, and feature tracking. This review evaluates the role of CMR as a risk stratification tool in DCM patients referred for ICD.

## 1. Introduction

The indications for primary prevention implantable cardioverter defibrillator (ICD) among patients with non-ischemic dilated cardiomyopathy (DCM) are a matter of controversy in light of the negative results of all randomized controlled trials [1,2,3]. There is a wide consensus concerning the lack of appropriate risk-stratification for ventricular arrhythmias (VA) and sudden cardiac death (SCD) as the main reason for the failure of the aforementioned trials.

In recent years, the improvement of risk stratification for VA and SCD has been the focus of intense research [4,5,6]. Cardiac magnetic resonance (CMR) will likely play a key role in the reassessment of risk-stratification in DCM, since late gadolinium enhancement (LGE) has consistently demonstrated a strong and independent association with VA and SCD in DCM [7,8,9,10,11]. In addition to LGE, other CMR techniques such as T1 mapping, extracellular volume fraction (ECV) quantification, left ventricular (LV) strain, or LV entropy may be useful to further characterize the arrhythmic risk in DCM. Finally, the application of artificial intelligence to CMR could enhance its pivotal role in the risk-stratification of patients with DCM.

## 2. Sudden Cardiac Death in Non-Ischaemic Dilated Cardiomyopathy

DCM is currently defined by the presence of LV or biventricular dilatation and systolic dysfunction in the absence of abnormal loading conditions (hypertension, valve disease) or coronary artery disease (CAD) sufficient to cause global systolic impairment [12,13,14,15]. The prevalence of DCM is estimated to be 1:2500 in the United States and around 30–40% of heart failure patients have a non-ischemic etiology [16]. DCM can be due to multiple etiologies, including genetic variants (30% of cases), drugs, toxics, hormonal abnormalities, and both infectious and non-infectious myocarditis [13].

It is estimated that SCD represents one third of cardiac deaths in DCM, however the risk of SCD is not uniform across the different etiologies of DCM [16]. In cases of DCM associated with pathogenic or likely pathogenic genetic variants in specific genes, such as lamin A/C (LMNA), filamin C (FLNC), transmembrane protein 43 (TMEM 43), phospholamban (PLN), desmoplakin (DSP), and RNA binding motif protein 20 (RBM20), the arrhythmic risk is higher [13,17,18,19,20,21].

It is also possible that risk factors for VA and SCD vary according to the etiology of DCM: in patients with LMNA, for example, non-sustained ventricular tachycardia (VT), male sex, left ventricular ejection fraction (LVEF) < 45%, non-missense variants, and atrioventricular (AV) block were associated with the arrhythmic risk and a specific risk calculator for LMNA variants carriers has been developed [22,23,24]. Similarly, in 2021 Verstraelen et al. proposed a new mutation-specific prediction model for individual VA risk in PLN p.Arg14del mutation [21].

## 3. The Controversy of Primary Prevention ICD in DCM

Although ICDs are effective in the prevention of SCD, randomized trials have shown a significant survival benefit with primary prevention ICD in DCM (Table 1). Current recommendations for primary prevention ICD indications are based on the results of a meta-analysis, which found a significant reduction in mortality with primary prevention ICD in DCM by combining the results of all randomized trials [25]. It should be noted that all randomized trials of primary prevention ICD in DCM used LVEF ≤ 35% as the main inclusion criterion. However, if used alone, LVEF is neither specific nor sensitive for SCD. On the one hand, as shown, for example, in the Maastricht registry, severe LV dysfunction is not present in the majority of patients who die from SCD [26]. On the other hand, only a minority of patients with DCM implanted with a primary prevention ICD due to severe LV dysfunction will actually receive appropriate ICD therapies. For example, in the DANISH trial, during a median follow-up of 68 months, only 11.5% of patients randomized to ICD received an appropriate shock to treat fast ventricular tachycardia or ventricular fibrillation [3]. Therefore, a major reason for the failure of the aforementioned trials has been the inability to select patients at high risk for SCD. Another factor that influences the potential benefit of primary prevention ICD is the competing risk of non-sudden death; for example, the DANISH trial showed a significant reduction in mortality with ICD only among those ≤70 yo, clearly because those >70 yo have a greater risk of competing lethal events [27].

Despite the improvements in materials and techniques, the implantation of ICD/cardiac resynchronization therapy (CRT) is associated with the risk of potential complications such as lead dislocation, infections, and inappropriate shocks; this risk increases in parallel with the complexity of the procedure [28,29,30].

## 4. Ventricular Arrhythmias and Sudden Death Risk Stratification in DCM

An effective risk stratification for SCD in DCM is essential but, at the same time, extremely difficult. In the past, several studies focused on electrocardiographic (including signal averaged electrocardiogram-ECG) and echocardiographic parameters; these results have been summarized in the meta-analysis performed by Goldberger et al. in 2014. This comprehensive analysis revealed that a depolarization parameter, fragmented QRS, and a repolarization parameter, T wave alternans, showed the highest odds ratio (OR) for identifying the arrhythmic events (OR: 6.73 and 4.66, respectively) and that LVEF showed a lower odds ratio when compared with the other analyzed functional parameter, the LVED dimension (OR: 2.87 vs. 3.47). Additionally, LVEF showed 71% sensitivity and 51% specificity for SCD, suggesting that LVEF, if used alone, is a relatively weak event predictor.

In the absence of a single very powerful predictor, the meta-analysis concluded that the risk stratification of SCD might improve with the use of multiple marker models. In fact, it has been shown that a high level of discrimination (OR ranging 15 to 20) would be warranted in order to correctly stratify for clinical purposes, and, thus, only a combination of different parameters may provide such high prediction levels. In this regard, the modest prediction ability of each marker included in the meta-analysis may reflect the prerequisite that a pathophysiological interplay among different factors may be required to get to the fatal epilogue of the SCD [31].

More recently, new predictors of VA and SCD have been studied. Among them, we highlight genetic variants in high-risk genes (e.g., LMNA, FLNC, TMEM43, PLB, DSP, RBM20), inflammatory mediators (e.g., high-sensitivity C-reactive protein-hsCPR), and tissue characterization markers (e.g., LGE, grey zone) (Table 2).

## 5. LGE and the Risk of Ventricular Arrhythmias and Sudden Cardiac Death

**Figure 1:**
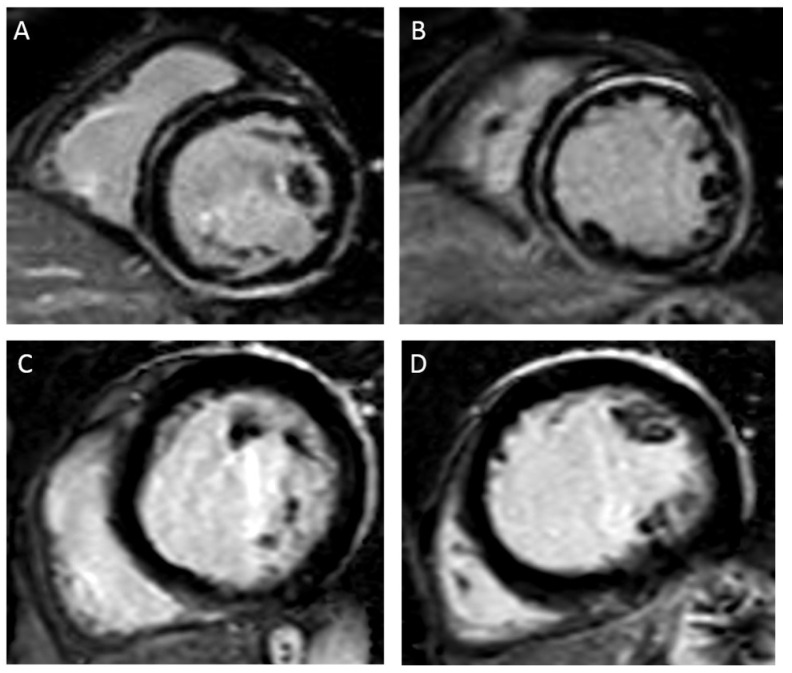
(**A**,**B**) show short axis views of the basal and mid segments in late gadolinium enhancement (LGE) sequences in a patient with non-ischemic cardiomyopathy and LVEF 40%. Extensive LGE (mid-wall septal and subepicardial) is observed. This patient had sustained monomorphic VT and underwent successful epicardial VT ablation. (**C**,**D**) show short axis views of the basal and mid segments in LGE sequences in a patient with non-ischemic cardiomyopathy and LVEF 27%. No LGE is observed. This patient has never experienced ventricular arrhythmias during follow-up.

Late gadolinium enhancement (LGE) is a technique used with the purpose of myocardial tissue characterization and, in particular, to identify localized myocardial fibrosis through the employment of gadolinium, a paramagnetic contrast agent. Gadolinium is injected intravenously, spreads outside the intravascular space, and can (hyper)enhance the tissue by shortening T1 (Figure 1). In this process, some variables, such as the regional distribution pattern within the extracellular space, wash-in and wash-out velocity, and the membrane cells’ integrity, become relevant [32,33]. Therefore, LGE derives from different concentration of gadolinium depending on local kinetic variability correlated to the different tissue representations (e.g., myocardial edema, necrosis, collagen deposition, or exogenous material accumulation) [34].

The prevalence of LGE in DCM ranges from 26% to 56% [35,36]. Only one study demonstrated a very high prevalence of LGE (71%) with a midwall distribution and less commonly epicardial pattern [37].

Myocardial scarring is recognized as the main substrate for sustained monomorphic ventricular tachycardia, since it provides all the elements that allow the maintenance of a reentry circuit. This has been demonstrated in cases of ischemic cardiomyopathy with prior myocardial infarction, but it has also been confirmed in patients with DCM who, during electroanatomic mapping, often display scars harboring VT circuits [38,39].

**Figure 2:**
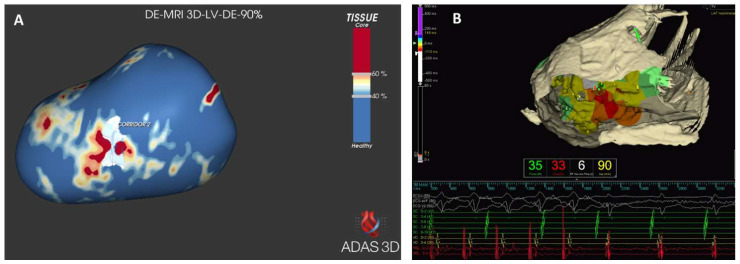
Functional correlation between the scar identified with late gadolinium enhancement and the ventricular tachycardia circuit in a patient with non-ischemic cardiomyopathy. In (**A**), CMR images are reconstructed with the ADAS 3D software (ADAS 3D Medical, Barcelona, Spain, https://www.adas3d.com) to identify border zone corridors. In this case one corridor is identified in the subepicardium of the mid-lateral left ventricular wall. This corridor nicely matches with the critical isthmus of the sustained monomorphic tachycardia induced during the ablation procedure, shown in (**B**). Ablation inside the isthmus determined the interruption of the ventricular tachycardia after 6 s (**B**).

The role of LGE as a predictor of VA and SCD was investigated in several observational studies [40,41]. From the outset, LGE has been shown to be an independent predictor of SCD and VA [42]. Subsequently, larger studies confirmed the association between LGE and major arrhythmic events, including after adjustment, for other clinical and functional parameters [37] (Figure 2). Gulati et al. showed that in 142 patients with DCM the arrhythmic endpoint (SCD, appropriate shocks ICD, non-fatal VT/VF) was achieved in 29.6% of patients with midwall LGE compared to 7% of those without LGE at a median follow up of 5.3 years and that the combination of LGE with LVEF allowed a better reclassification of patients at high and low arrhythmic risk [43]. Another study on 175 patients followed up for a median of 5 years detected LGE in 70% of them and showed that the presence of septal and lateral midwall LGE was strongly associated with life-threatening VA (HR 23.1) [44].

Following updated international recommendation of ICD implantation in primary prevention in 2015, more studies appeared in the literature, some of those enrolling patients with no indication to the prophylactic therapy. Halliday et al. first demonstrated an association between LGE and arrhythmic events in patients with LVEF > 40%. They showed that their population with non-ischemic DCM, moderate-to-mild reduction of LVEF, and presence of midwall LGE had a risk of major arrhythmic events, similar to that of other published cohorts of DCM patients with severely depressed LVEF and without evidence of LGE (approximately 3.6%/year) [45].

Recently, Di Marco et al. considered patients with non-ischemic DCM and a wide spectrum of LVEF. LGE was an independent and very strong predictor of VA and SCD across all LVEF strata and high-risk LGE distributions were identified, such as epicardial or transmural LGE or combined septal and free wall LGE. The authors generated a new risk stratification model that identified a very low risk group (0.2% events/year), LGE negative and LVEF ≥ 20%, a high risk group (7.2% events/year), LGE positive and LVEF ≤ 35%, and intermediate-high risk group (2.8% events/year) in case of presence of high risk LGE distribution and LFEF > 35% [10]. Therefore, the presence of specific patterns of LGE may confer a higher risk of events irrespective of LVEF.

The significant and strong association between LGE and VA or SCD has also been confirmed in metanalysis. [46].

Further consideration is required for patients undergoing CRT where LGE was demonstrated to be able to predict arrhythmic events. In an observational study enrolling 252 patients with DCM and CRT, of whom 68 had LGE, it was observed that CRT-D was associated with significantly higher survival than CRT-P only in patients with LGE. In patients without LGE, with their low arrhythmic risk, CRT-D offered no benefit compared with CRT-P [47].

## 6. Extension of Late Gadolinium Enhancement and Association with Ventricular Arrhythmias

The literature presents diverging results regarding the relationship between LGE extension and arrhythmic risk [46,48]. Some studies have shown a relationship between the extent of LGE and the risk of SCD, VA, and cardiovascular death [43,49,50,51]. Furthermore, some have shown that the extent of LGE was more predictive than the presence of LGE alone [9,42,52].

Recently, Li et al. showed that a myocardial scar greater than or equal to 7.1% of the LV mass is associated with SCD or aborted SCD [53]. Klem et al. demonstrated a curvilinear relationship between risk of arrhythmic events and scar size on LGE, reaching a plateau at 20% to 25%, regardless of LVEF. Moreover, in that cohort, a relatively small scar extent of 2.0% provided the optimal threshold for prediction of SCD in patients with LVEF ≤ 35% and >35% [54]. Furthermore, there is evidence of a nonlinear relationship between adverse outcomes and LGE extension. Both in 2017 and 2019, Halliday et al. highlighted that the percentage extent of LV LGE predicting the arrhythmic endpoint (SCD and aborted SCD) was 0% and 0.71%, respectively, with small amounts of LGE predicting a substantial increase in risk [45,55]. Similarly, Perazzolo Marra et al. revealed a significant correlation between the LV-LGE presence and major arrhythmic events, not affected by the amount and distribution [36]. Therefore, not all authors support the predictive value of LGE extension for SCD and VA as a linear relationship, and specific cut-off values are still lacking [56].

## 7. Location/Pattern of Late Gadolinium Enhancement and Association with Ventricular Arrhythmias

In addition to the presence and extension of LGE, the localization and pattern were studied. The most frequent patterns in DCM are subepicardial, linear midwall, patchy, or transmural that do not follow a coronary territory and the concomitant evidence of multiple LGE pattern types (mid-wall striae or patches, sub-endocardial, or sub-epicardial enhancement) increases the risk of all-cause mortality [7,57]. Some studies have shown that patients with midwall LGE had an increased risk of SCD and appropriate shocks of the ICD; in others, this correlation was seen with subepicardial LGE [35,42,43,44,51,58,59,60,61,62,63]. In the study of Halliday et al. LGE distribution proved to be superior to its presence, extension, or pattern and combined presence of septal and free-wall LGE was associated with a high arrhythmic risk. Additionally, sub-epicardial or multiple patterns of LGE were associated with a high-risk of SCD events [55].

In line with these findings, Di Marco et al. observed that the presence of epicardial LGE, transmural LGE, or combined free-wall and septal LGE were associated with higher arrhythmic risk compared with other LGE distributions or LGE absence, improving the risk stratification for VA and SCD, especially for patients with LVEF > 35% [10].

Interestingly, LGE is rare in patients with low-risk genetic variants, while patients with variants at greater arrhythmic risks present more typical patterns: DSP, FLNC, and PLN with a predominance of LGE subepicardial ring-like scar pattern, LMNA with a mid-wall basal, or septal LGE distribution, whereas titin (TTN), BAG cochaperone-3 (BAG3), duchenne muscular dystrophy (DMD), RBM20, and some form of LMNA genotypes show unspecific or heterogeneous LGE patterns [13,64,65,66].

However, not all studies agree on the correlation between the septal/free wall localization or the subepicardial/mid-wall distribution of the LGE and increased arrhythmic risk [36,44,49,51,57,59,62,67].

Although there are conflicting data, the most recent evidence suggests that imaging quantification and localization of myocardial fibrosis via CMR LGE represents a strong predictor of major malignant arrhythmic events in patients with DCM. Large cohort studies, preferably combining CMR information with other clinical data (genetic testing in the first instance) are required to create a more individualized DCM management approach.

## 8. Insertion Points

LGE can be also localized to the anterior and posterior right ventricular insertion points (RVIP). Limited studies that did not specifically focus on this type of LGE localization showed conflicting results on the outcome of these patients [49,68]. In the study of Yi et al., isolated localization on RVIP was associated with a lower LGE extension and did not significantly increase adverse arrhythmic events compared to the patients without LGE [69]. These findings have been confirmed by Claver et al. in a large cohort study that showed how patients with DCM and LGE at RVIP have a low arrhythmic and SCD risk compared to patients with other LGE distribution. Interestingly, patients with LGE at RVIP had significantly lower RVEF and both higher indexed RV end-diastolic and end-systolic volumes, suggesting that this peculiar LGE localization may be a consequence of RV pressure overload [70].

## 9. Limitations of Late Gadolinium Enhancement

One of the main limitations of LGE is linked to the magnetic resonance technique, specifically in that there are long acquisition times, high costs, and the contraindication of the use of contrast in subjects with renal insufficiency, which is often present in patients with DCM [71].

The second limit is that quantitative LGE evaluation is not a standardized technique. It results in heterogenicity of the methods for evaluating and quantifying LGE, such as number of segments, percentage, or absolute weight with no defined cut-off values [37,43,49,52,72,73,74,75,76].

Some studies used semi-quantitative evaluations. Guaricci et al., in a registry to evaluate the additional prognostic value of a composite CMR-based risk score over standard-of-care in a large cohort of consecutive unselected non-ischaemic DCM patients, performed a semi-quantitative analysis evaluating the presence of LGE in a segment of the 17-segment model [8].

Another limit of LGE is that although the presence and extent of LGE on CMR is a good predictor for monomorphic VT, it is less specific for potentially fatal polymorphic VT/VF [52].

Lastly, LGE reflects only focal fibrosis, but also some patients with diffuse interstitial fibrosis experienced VA events and SCD [77]. Therefore, additional CMR parameters could be necessary, such as T1 mapping technique.

## 10. T1 Mapping and Extracellular Volume Quantification

T1 mapping and ECV are both important techniques used in CMR to assess the structure and function of the heart in detail [78]. T1 mapping is a technique that measures the longitudinal relaxation time (T1) of tissue [78]. T1 is the time it takes for a tissue to return to its state of magnetic equilibrium after being perturbed by radiofrequency [78]. Different types of tissue have different T1 values, making it possible to distinguish and characterize tissues according to their magnetic properties [78]. In the cardiac context, T1 mapping is useful for assessing changes in cardiac tissue composition; it can identify and quantify various conditions such as myocardial fibrosis, inflammation, oedema, and fat accumulation in the myocardium [78]. ECV is a measure of the volume fraction of the extracellular space relative to the total tissue volume [78]. ECV and T1 mapping allow for quantification of diffuse fibrosis, providing complementary information to that of LGE [78].

Tissue inhomogeneity caused by diffuse fibrosis and cellular disarray is a potential substrate for the initiation of life-threatening ventricular arrhythmias. However, the pathophysiological mechanisms underlying arrhythmogenicity resulting from diffuse or focal fibrosis are still poorly understood and further studies are needed [79].

CMR parametric mapping techniques allow us to evaluate diffuse fibrosis even in the absence of LGE; changes in T1 values may occur in the early stages of DCM when the LVEF is only slightly reduced [48,80,81]. The importance of T1 mapping is emphasized in diseases such as Anderson Fabry disease and Cardiac Amyloidosis, where these values are reduced or increased. T1 mapping can therefore be a red flag, pointing the clinician towards a specific etiological diagnosis prior to the development of LGE [82,83,84,85].

Puntmann et al. showed that the native T1 had a sensitivity of 100% and specificity of 97% to discriminate a healthy myocardium from a diseased one. Both T1 mapping and ECV are associated with all-cause mortality and HF in patients with DCM [86]. In a recent study, the potential predictive value of quantitative CMR features for Major Adverse Cardiac Events (MACEs) was explored in patients diagnosed with DCM. It was observed that DCM patients who experienced heart failure or arrhythmia-related events exhibited significantly higher levels of both native T1 and ECV compared to patients who did not experience MACEs [87].

In a study by Chen et al., native T1 mapping was independently associated with sustained VT and appropriate ICD shocks, and this association persisted even after LGE correction [88]. In another study, subjects with DCM and a history of complex VA had a higher native T1 than those without a history of VA and this association persisted even after adjustment with LVEF and LGE [79]. As for the ECV, it has been shown to be significantly associated with a combined endpoint of cardiovascular mortality, hospitalization for HF, and appropriate ICD shocks in a cohort of 89 DCM patients, even after adjustment for LVEF [89]. However, such combined endpoints, including heart failure (HF) and arrhythmic events at the same time, does not allow us assess the specific association between LGE and VA or SD. Finally, in a study of 240 patients with DCM, it was found that ECV was, together with LVEF, the only CMR parameter independently associated with a combined endpoint of death from any cause or hospitalizations for heart failure [90]. The association of ECV with heart failure events in this study is a warning with respect to the potential specific association between ECV and VA or SD. A subsequent analysis of data from a single-center prospective registry of 618 nonischemic cardiomyopathy (NICM) patients with available ECV data showed that mean ECV was significantly associated with the combined primary endpoint (which included appropriate implantable cardioverter defibrillator therapy, sustained ventricular tachycardia, resuscitated cardiac arrest, and SD) while native T1 was not an independent predictor of the arrhythmic endpoint. A cut-off of ECV ≥ 30% was the strongest independent predictor of the primary endpoint (HR 14.1, *p* = 0.01) after adjustment for LGE and LVEF. ECV ≥ 30% discriminated arrhythmic risk between LGE+ cases and those with LVEF ≤ 35%. A simple clinical risk stratification model based on LGE, LVEF ≤ 5%, and ECV ≥ 30% achieved excellent predictive power (Harrell’s C 0.82) and reclassified 32% of the study population from LVEF ≤ 35% alone [91]. Based on the previously cited studies, the use of T1 mapping and, above all, ECV calculation are promising tools to further improve the risk stratification for VA and SD in DCM on top of LGE [79]. However, more studies are needed to understand the exact role of these parameters in the risk stratification for VA and SD and to find the optimal cut-offs to discriminate patients at high vs. low risk.

## 11. Limitations of ECV and T1 Mapping Quantification

While T1 mapping and ECV quantification are powerful techniques in CMR imaging, they have certain limitations. The limitations concern the data acquisition, post-processing, and interpretation phases. One notable challenge is the potential for measurement variability due to factors such as heart rate, magnetic field strength, and the specific CMR protocol used. In addition, the presence of motion artefacts or inadequate breath holding during image acquisition can lead to inaccuracies in T1 mapping and ECV calculations. For acquisition, the mapping technique can vary depending on the amount and speed of contrast injection and the time between contrast injection and T1 mapping acquisition. For post-processing, the most commonly used technique is the single slice over the middle ventricle, which may not adequately represent inhomogeneous fibrosis [60]. It is also important to note that T1 mapping and ECV values can be influenced by factors other than myocardial fibrosis, such as oedema, inflammation, and infiltrative disease. Furthermore, standardization of techniques and reference ranges for T1 mapping across different CMR platforms and centers is an ongoing area of research. Finally, it is not always easy to distinguish subjects with DCM from normal subjects on the basis of T1 values due to the presence of borderline data [81]. ECV has the advantage over T1 of being more reproducible and suitable for providing cut-offs that can be universally validated and used [92]. Despite these limitations, when used judiciously and interpreted in conjunction with the clinical context, T1 mapping and ECV quantification remain invaluable tools for assessing myocardial tissue characteristics in various cardiac pathologies.

## 12. Assessment of Strain via CMR

Ejection fraction describes the overall myocardial function of the left ventricle, while strain imaging allows evaluation of regional myocardial deformation and can detect myocardial dysfunction before ejection fraction decreases. The most used magnetic resonance imaging technique to evaluate strain is MR feature tracking (MR-FT) and the cut-off values vary depending on the software, modality, and methods [93,94]. With MR-FT, it is possible to quantitatively assess the contractility and deformation of the heart walls before the ejection fraction decreases. A retrospective observational study involving 161 patients with DCM demonstrated that CMR-FT measurements of three-dimensional myocardial strain parameters, specifically Global Longitudinal Strain (GLS) and Global circumferential Strain (GCS), held certain diagnostic value and were capable of reflecting the underlying abnormality of ventricular mechanics in DCM with microvascular angina (MVA) [95]. Altogether, GLS is associated with total mortality and cardiac events in heart failure with preserved ejection fraction (HFpEF).

Buss et al. evaluated 210 patients with DCM and observed that GLS and LGE mass were the only independent predictors of a combined endpoint including cardiac death, heart transplant, and appropriate ICD shock. The best cut-off of GLS to predict the combined outcome was −12.5%: this cut-off significantly discriminated the prognosis in all subgroups analyzed (those with LVEF < 35%, those with LVEF > 35%, those with LGE, and those without LGE) [96]. Another study, which included 507 DCM patients, showed that GLS was an independent predictor for death from any cause, after adjustment for LGE and LVEF [97].

However, in the previously mentioned cohort of 618 patients with NICM and comprehensive CMR evaluation, GLS showed high collinearity with LVEF and lost its association with the arrhythmic outcome after adjustment for LVEF [91].

In summary, strain analysis with CMR may provide relevant prognostic contribution with respect to the overall prognosis. However, it is not clear whether CMR-based strain parameters can improve the risk stratification for VA and SCD on top of LVEF and LGE. Technically, MR-FT’s advantage over LGE and T1 mapping lies in its ability to evaluate MVA without the need for contrast media, making it especially pertinent for patients with contraindications to gadolinium-based agents. Additional studies are needed to see if GLS can add prognostic value for VA and SD on top of LGE and of markers of diffuse fibrosis.

## 13. Limitations of Strain

Despite its undoubted usefulness, feature tracking in CMR has certain limitations. The first limitation of the MR-FT is related to the low temporal resolution, which can underestimate the strain values. Furthermore, the cut-off values vary by software and method and this makes comparison between studies difficult [98]. Finally, the radial and segmental strain values still lack reliability [93].

## 14. Assessment of Myocardial Heterogeneity Derived from CMR

Entropy evaluates the probability of distribution of myocardial pixel signal intensity and is therefore an MRI-derived measure of myocardial heterogenicity. Rahul et al. evaluated 130 patients with DCM who received a primary prevention ICD and observed that LV entropy was an independent predictor of VA and SCD but had no significant independent association with a combined heart failure endpoint including cardiac death, heart transplantation, and left ventricular assist device implant [99]. This is an interesting technique and further studies are necessary to clarify its role in risk prediction in DCM.

## 15. Artificial Intelligence Applied to CMR

**Figure 3:**
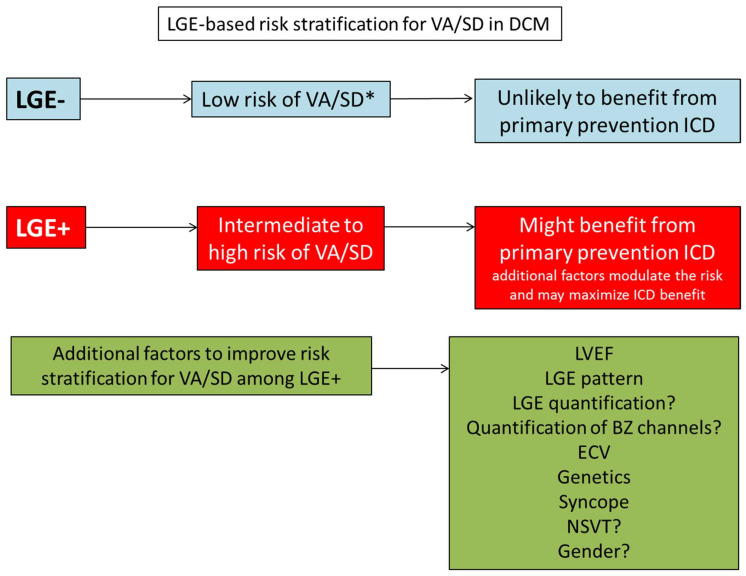
Schematic representation of a potential risk-stratification and ICD selection strategy based on LGE. Additional factors may have variable prognostic weight in different sub-population of LGE + DCM patients. Risk factors for non-sudden death, such as Age, NYHA class and comorbidities will also influence ICD benefit. LGE: late gadolinium enhancement. VA: ventricular arrhythmias. SD: sudden death. ICD: Implantable cardioverter defibrillator. LVEF: left ventricular ejection fraction. ECV: extracellular volume fraction. NSVT: non sustained ventricular tachycardia. * LGE− patients with very severe LV dysfunction (LVEF ≤ 20%) have been suggested to have higher risk of VA/SD; however, these patients might have a not negligible competing risk of non-sudden death with a direct impact on the potential benefit of primary prevention ICD.

Artificial intelligence (AI) aims to develop computers with human intelligence, which include machine learning (ML) and deep learning (DL), and has emerged as one of the main innovations in the field of cardiovascular diagnostic imaging [100,101,102] (Figure 3). ML and DL techniques could achieve a more standardized quantification of LGE by overcoming the limits related to its irregular and multifocal appearance, and to the variability between centers in accuracy and reproducibility [103,104,105,106,107]. ML can also be applied for T1 mapping and ECV, allowing for the assessment of adverse events in patients with mildly or moderately depressed LVEF [100,108,109]. Chen et al. evaluated a model for predicting cardiovascular events in patients with DCM based on ML obtained from patient baseline characteristics, blood tests, ECG, echocardiography and CMR [110]. Artificial intelligence techniques therefore could offer a better appreciation of the phenotypic heterogenicity of DCM patients with implications in risk stratification, early detection, and personalized therapies [111].

## 16. Future Direction

Additional studies are needed to evaluate the role of CMR parameters other than LGE, with a special focus towards those related with the evaluation of diffuse fibrosis. Further studies should focus on LGE+ patients, to distinguish those with the highest arrhythmic risk by combining LGE characteristics, ECV, genetics and other potential predictors [112]. The large body of evidence supporting the association between LGE and VA and SCD would already justify the realization of randomized controlled trials to evaluate the non-inferiority of medical treatment vs. primary prevention ICD among patients considered to be at low arrhythmic risk, such as patients with LVEF ≤ 35% without LGE. Actually, two LGE-based randomized trials are already recruiting: the CMR-ICD study (NCT04558723) will randomize to ICD or medical therapy 760 patients with DCM, LVEF ≤ 35% and LGE and the CMR-GUIDE trial is randomizing to ICD or medical therapy 428 patients with LGE and LVEF > 35% of both ischemic and non-ischemic etiology [113].

Larger, prospective studies evaluating other CMR parameters in SCD risk stratification among patients with DCM are lacking [114]. To address gaps in prognostic stratification, we should take into account all DCM spectrum, irrespective of LVEF.

Finally, the development and use of artificial intelligence and machine learning techniques applied in the prognostic stratification of patients undergoing CMR will become increasingly important, providing the clinician with a crucial tool to use in the clinical practice decision-making.

## 17. Conclusions

Predicting the risk of SCD is one of the most difficult challenges in the cardiovascular field despite numerous efforts to identify patients who can benefit from prophylactic ICD therapy. This is because arrhythmic risk is multifactorial and related to genetic, acquired, anatomical and pathophysiological factors. The scientific literature supporting the possibility of improving the prediction of an individual patient’s arrhythmic risk by means of cardiac magnetic resonance imaging is growing in number. Currently, cardiac MRI can improve risk stratification by exploiting cine sequences, tissue characterization including LGE, T1 and T2 mapping, ECV calculation and feature tracking. A reasonable hope of further prognostically useful information could come from opening up new frontiers of development and in particular from artificial intelligence.

## Figures and Tables

**Table 1:** Clinical Trials on ICD use in primary prevention.

	SCD-HeFT	DEFINITE	DANISH	AMIOVIRT	CAT
Year	2005	2004	2016	2003	2002
Design	ICD versus amiodarone versus OMT	ICD versus OMT	ICD versus OMT	ICD versus amiodarone	ICD versus OMT
Inclusion criteria	LVEF < 35% NYHA II–III	LVEF < 36% NYHA I–III NSVT or PVCs	LVEF < 35% NYHA II–III (IV if CRT) NT-proBNP > 200 pg/mL	LVEF ≤ 35% NYHA I–III NSVT	LVEF < 30% NYHA II–III
% DCM	47	100	100	100	100
Mean EF%	25 ± 5	21 ± 14	25	23 ± 9	24 ± 7
All-cause mortality (only in DCM group)	ICD 21.4%; OMT 27.9% (5 years) HR 0.73; 95% CI 0.50 to 1.07; *p* = 0.06	ICD 12.2%; OMT 17.4% HR 0.65; 95%CI 0.40 to 1.06; *p* = 0.08	ICD 21.6%; OMT 23.4% HR 0.87; 95% CI 0.68 to 1.12; *p* = 0.28	Terminated early	Terminated early
SCD	Not applicable	ICD 1.3%; OMT 6.1% HR 0.20; 95%CI 0.06 to 0.71; *p* = 0.006	ICD 4.3%; OMT 8.2% HR 0.50; 95% CI 0.31 to 0.82; *p* = 0.005		Not applicable

DCM: dilated cardiomyopathy. SCD: sudden cardiac death. ICD: implantable cardioverter defibrillator. OMT: optimal medical therapy. LVEF: left ventricular ejection fraction. NYHA: New York Heart Association. PVC: premature ventricular contraction. CRT: cardiac resynchronization therapy. NT-proBNP: N-terminal pro-brain natriuretic peptide. NSVT: non-sustained ventricular tachycardia. HR: hazard ratio. CI: confidence interval.

**Table 2:** CMR parameter for risk stratification of SCD and their limitations.

	Characteristics	Limitations
LGE	Evidence of myocardial scar extension, pattern and localization as risk predictor for VA/SCD	Contraindication of the use of contrast in renal insufficiency Heterogenicity of the methods for evaluating and quantifying LGE Limited predictive power for VF/polymorphic VT
T1 mapping/ECV	Quantification of myocardial fibrosis, oedema and fat accumulation Use regardless renal function Higher native T1 values are associated with arrhythmic events	Measurement variability due to heart rate, magnetic field strength and specific CMR protocol Data acquisition susceptibility to motion artefact, inadequate breath holding, amount and speed of contrast injection T1/ECV values influenced by oedema, infiltrative disease and inflammation
Strain imaging	Evaluation of regional myocardial dysfunction and deformation	Underestimation due to low temporal resolution Cut-off values variability Lack reliability of the radial and segmental strain values

LGE: Late gadolinium enhancement. ECV: extracellular volume. VA: ventricular arrhythmias. SCD: sudden cardiac death. VF: ventricular fibrillation. VT: ventricular tachycardia. CMR: cardiac magnetic resonance.

## Data Availability

No new data were created for this review.
